# Thirty-year survey of bibliometrics used in the research literature of pain: Analysis, evolution, and pitfalls

**DOI:** 10.3389/fpain.2023.1071453

**Published:** 2023-03-01

**Authors:** Claude Robert, Concepción Shimizu Wilson

**Affiliations:** ^1^ Gliaxone, Saint Germain sous Doue, France; ^2^ School of Information Systems, Technology and Management, University of New South Wales, UNSW Sydney, Sydney, NSW, Australia

**Keywords:** pain, pain literature analysis, bibliometrics, gender publishing profile, scientometrics, software visualization tools, publishing pitfalls

## Abstract

During the last decades, the emergence of Bibliometrics and the progress in Pain research have led to a proliferation of bibliometric studies on the medical and scientific literature of pain (B/P). This study charts the evolution of the B/P literature published during the last 30 years. Using various searching techniques, 189 B/P studies published from 1993 to August 2022 were collected for analysis—half were published since 2018. Most of the selected B/P publications use *classic* bibliometric analysis of Pain *in toto*, while some focus on specific types of Pain with Headache/Migraine, Low Back Pain, Chronic Pain, and Cancer Pain dominating. Each study is characterized by the origin (geographical, economical, institutional, …) and the medical/scientific context over a specified time span to provide a detailed landscape of the Pain research literature. Some B/P studies have been developed to pinpoint difficulties in appropriately identifying the Pain literature or to highlight some general publishing pitfalls. Having observed that most of the recent B/P studies have integrated newly emergent software visualization tools (SVTs), we found an increase of anomalies and suggest that readers exercise caution when interpreting results in the B/P literature details.

## 1. Introduction

During the last decades, research on pain has made massive progress resulting in an explosion of scientific and medical publications.

This increase in publications was accompanied by a continuous restructuring not only of the intellectual input, but also of the dissemination output of research on pain. Some illustrative examples include:

• the recent emergence of journals dedicated either to pain in general (e.g., *Neurobiology of Pain* launched in 2016, *Pain Reports* in 2016 and *Frontiers in Pain Research* in 2020), or to specific pain topics (e.g., *The Journal of Dental Anesthesia and Pain Medicine* launched in 2015 and *Paedriatric and Neonatal Pain* in 2019);
• the creation of associations (e.g., the *American Association of Pain Psychology*, AAPP formed in 2010 and the *US Association for the Study of Pain*, USASP in 2019—the latter purchasing the *Journal of Pain* in 2020); and
• the addition of Special Interest Groups (SIGs) and Chapters to existing associations (e.g., the Neuropathic Pain SIG or NeuPSIG and the Chinese Association for the Study of Pain or CASP, a Chapter of the International Association for the Study of Pain, IASP).

Although of lesser importance quantitatively, another factor explaining the increase in the number of publications is the explosion in the use of bibliometrics in specific literatures (e.g., pain) to design a set of quantitative methods for the analysis of scientific publications (1). This publication growth can be illustrated in a search of the *PubMed* database using the MeSH (Medical Subject Headings) term, *Bibliometrics*, which retrieved nearly 13,000 publications distributed over 30 years in three 10-year periods: 623 publications from 1991 to 2000; 3,677 from 2001 to 2010; and 8,641 from 2011 to 2020. Additionally, more recent bibliometric papers in the biomedical field range over numerous topics such as cancer (2, 3), radiology (4, 5), and coronavirus (6, 7). In this context, the objective of the utilization of bibliometrics varies from charting the growth and development of a research field (8); to evaluate the progress of a researcher (9), an institution (10), countries (11), or a journal (12); or to provide statistics to support science decisions, research policies and collaborative research initiatives (13).

Looking at the numerous bibliometric studies, what may appear as a duplication of bibliometric studies on the same subject can be a source of confusion or misunderstanding if one is not fully aware of the different parameters used in each study such as: the time span analyzed, the database(s) used, the criteria for inclusion of papers or, whether a general or specific aspect of a subject is targeted, etc. Readers may negate or misinterpret the results of the bibliometric approach, perhaps due to a lack of understanding of the subject of bibliometrics leading to a lack of confidence in the reliability of the study.

Considering these obstacles and given the increasing number of bibliometric studies on pain, our objective is to present a bibliometric study on existing pain papers using various bibliometric techniques to highlight some general points, both in the methodologies used and the objectives pursued. Based on an analysis of these papers, our aim is to provide the reader with some information and understanding to enable a better appreciation of the content of these studies, and thus be better prepared for the reading and analysis of future bibliometric studies on pain and other related literatures. In our approach, papers are sorted and analyzed according to their goals: either to provide a general, detailed, or specific topic description of the pain literature (or specific pain subtopics), or to investigate (or highlight) some characteristics of the publication process, not just applicable to the bibliometrics of the pain literature, but generally to the bibliometrics of all scientific and medical subject literatures.

## 2. Methods

The process developed from March 2021 to August 2022 is as follows:

• A *PubMed* search was conducted with one of the following keywords “bibliometrics, scientometrics, informetrics” associated with one of the following pain-related keywords “pain, nociception, analgesia, headache, migraine, cephalalgia”. Each term was truncated appropriately to retrieve variant word forms. These keywords, (considered as the basis of the pain scientific terminology) were searched in the titles, abstracts, or keywords of documents. The resulting documents (mostly papers or articles), with no language restriction, were further scrutinized by the two authors and retained if their contents are in line with the stated objective.
• The same procedure was performed on the *Web of Science (WoS)*, *Scopus*, *ProQuest*, and *Google Scholar*.
• The reference list of each paper was analyzed to capture possibly missed papers, and their citations (obtained using *Google Scholar*) were scrutinized to capture any additional missed papers.
• Finally, a “random” search was conducted on general search engines such as *Google*, *Bing*, *Qwant* using additional pain and bibliometric terms such as “trigeminal neuralgia”, “low back pain”, “literature analysis”, “quantitative scientific literature”. Papers not retrieved earlier were added to our dataset.

This method can appear as an “unusual/unorthodox” way of searching, but we think it is well-adapted to our topic relating to the bibliometric analysis of the literature related to pain (B/P for short) for which using only the main descriptors “Bibliometrics” and “Pain” in a typical search strategy would not be adequate to capture the targeted literature and/or would attract too many irrelevant papers. Additionally, this strategy will, most likely, miss very few papers.

In order to structure the analysis, each paper was classified in one of three categories: (A) *General Purpose*—where the aim of the paper is to provide an overview of a general or a specific topic (e.g., historical approach, specific type of pain) of the pain literature; (B) *Non-Specific*—where papers highlight the publishing characteristics or pitfalls of the pain literature using bibliometric analysis; and (C) *Miscellaneous*—where papers could not be assigned to one of the previous categories.

## 3. Results and discussion

From our various search strategies, 189 publications on B/P were deemed relevant and formed our dataset for analysis.

The earliest paper was published in 1993 and none appeared until 1999 onwards. The first two 5-year periods (1998–2002 and 2003–2007) were relatively stable with 7 and 8 papers, respectively; however, since then the numbers of B/P papers exploded: 19 papers in 2008–2012, 34 in 2013–2017, 115 papers from 2018 to August 2022, and three papers are “under review” or “in press”.

The first authors of the 189 papers were from 30 countries, distributed in descending order of productivity: China (69 papers), the USA (35 papers), Brazil (13 papers), Canada and Spain each with 8 papers; South Korea (7 papers), Turkey (6 papers), France, India, and Italy each with 5 papers; Croatia (4 papers); and 19 countries with 3 or fewer B/P papers (Table 1).

**Table 1:** Distribution of first authors’ country affiliations in the bibliometrics of the pain literature.

Countries/Territories^a^	Number of papers
China	69
USA^a^	35
Brazil	13
Canada, Spain	8
South Korea	7
Turkey	6
France, India, Italy	5
Croatia	4
Switzerland^a^	3
Austria, Colombia, Cuba, Greece	2
Argentina, Australia, Bulgaria, Chile, Hungary, Iran, Mexico, Nigeria, Palestine, Philippines, Portugal, Sweden, Taiwan, Turkey	1

^a^ For one paper, the first author was affiliated with both Switzerland and the USA; hence counted twice.

When the main topic of each paper is considered, papers providing a general and *classical* bibliometric analysis of the pain literature (Group A) represented three-quarters (143 papers, 75.7%) of all the papers; those aimed at highlighting pitfalls in the publishing process (Group B) constituted 16.9% or 32 of the papers analyzed; and the rest in Group C (14 papers, 7.4%) are miscellaneous papers with objectives other than those in Groups A or B. Some general information is presented for each topic and for each publication (in chronological order) in Tables 2, 3—Group A, Table 4—Group B, and Table 5—Group C.

**Table 2:** (Group A) Selected variables of 143 documents (75.7%)—***general or classical*** bibliometric analysis of the pain (B/P) literature.

Ref.	Year of publication	Authors	Journal/Source of publication	Country affiliation of first author	Title of the study	Type of pain studied (as indicated by the authors)	Medical/Scientific context of study	Years covered	Database (s) searched	No. SVTs used
(14)	1993	Guardiola and Banos	*J Pain and Sympt Manage*	Spain	Is there an Increasing Interest in Pediatric Pain? Analysis of Biomedical Articles Published in the 1980s	n.r.	Pediatrics	1981–1990	Medline	0
(15)	1999	Norton et al.	*Pain*	Canada	Growing pain: 10-year research trends in the study of chronic pain and headache	Chronic pain	Limited to 6 specific topics	1986–1995	Medline, PsycLIT	0
(16)	1999	Strassels et al.	*Anesth Analg*	USA	Toward a Canon of the Pain and Analgesia Literature: A Citation Analysis	n.r.	n.r.	1981–1997	Institute for Scientific Information (ISI)	0
(17)	2000	Fernandez-Banea et al.	*Rev Soc Esp Dolor*	Spain	Importancia del dolor pediátrico en las publicaciones científicas	n.r.	Pediatrics	1998	Medline	0
(18)	2000	Gold and Roberto	*Geriatr Nursing*	USA	Correlates and Consequences of Chronic Pain in Older Adults	Chronic pain	Elderly	1990–1997	PubMed, CINAH, Psychlit, Psychfirst, Sociofile, SocioAbs, Ageline, Article first, Nursing/Allied Health, Social Gerodontology, UnCoVer	0
(19)	2001	Banos et al.	*Pain* and *Res Manage*	Spain	An analysis of articles on neonatal pain published from 1965 to 1999	n.r.	Pediatrics	1965–1999	PubMed	0
(20)	2002	Keefe et al.	*Psychosom Med*	USA	Changing Face of Pain: Evolution of Pain Research in Psychosomatic Medicine	n.r.	Psychosomatic Medicine	1939–1999	*Journal Psychosomatic Medicine*	0
(21)	2003	Goldenberg and Smith	*J Rheumatol*	USA	Fibromyalgia, Rheumatologists, and the Medical Literature: A Shaky Alliance	Fibromyalgia	n.r.	1982–2001	Medline	0
(22)	2003	Terajima and Aneman	*Acta Anesthesiol Scand*	Sweden	Citation classics in anaesthesia and pain journals: a literature review in the era of the internet	n.r.	n.r.	1986–2002	22 journals listed in anesthesiology (WoS)	0
(23)	2005	Gesztelyi and Bereczki	*Clin Neurosci/Ideggy Szle*	Hungary	The representation of headache in the Hungarian medical literature	Headache	n.r.	1950–2003	Clin Neurosci/Ideggyógyászati Szemle, Hungarian Medical Bibliography, Medline and Old Medline	0
(24)	2006	Turk	*Clin J Pain*	USA	Impact of Articles Published in the Clinical Journal of Pain: Most Frequently Cited Papers Published From 2002 to 2009	n.r.	n.r.	before 2002, 2006–2007	*Clin J Pain*	0
(25)	2007	Alonso et al.	*Rheumatol Clin*	Spain	A Bibliometric Approach to Research into Fibromyalgia	Fibromyalgia	n.r.	1980–2005	WoS: SCI-E, SSCI	0
(26)	2007	Friedberg et al.	*J Psychosom Res*	USA	Publication trends in chronic fatigue syndrome: Comparisons with fibromyalgia and fatigue: 1995–2004	Fibromyalgia	n.r.	1995–2004	OVID/Medline, PsycINFO + *J Chronic Fatigue Syndrome*	0
(27)	2007	Gomez and Conlee	*AATEX*	USA	An analysis of reporting pain and distress recognition and alleviation in scientific journal publications	n.r.	Pain in animals	2006–2007	39 journals	0
(28)	2008	Batista et al.	*Rev Gaúcha Enferm*	Brazil	Publicaçōes sobre dor e diagnóstico de enfermagem em uma base de dados brasileira	n.r.	Nursing diagnosis and intervention	until 2006	Brazilian nursing database (BDEnf)	0
(29)	2008	Marteen et al.	*Cephalalgia*	Switzerland, USA	Headache disorders in developing countries: research over the past decade	Headache	n.r.	1997–2006	PubMed and 66 other internationally accessible databases	0
(30)	2008a	Robert et al.	*Pain*	France	Bibliometric analysis of the scientific literature on pain research: A 2006 study	n.r.	n.r.	2006	CC Life Sciences/Clinical Medicine/Social and Behavioral Sciences (ISI-Thompson Scientific)	0
(31)	2008b	Robert et al.	*J Orofac Pain*	France	World Orofacial Pain Research Production: A Bibliometric Study (2004–2005)	Orofacial pain	n.r.	2004–2005	CC Life Sciences/Clinical Medicine (ISI-Thompson Scientific)	0
(32)	2009	Cocco	*Pathos*	Switzerland	A bibliometric approach to the Alternative Medicine in chronic pain	Chronic pain	n.r.	undetermined	PubMed	0
(33)	2009	Mogil et al.	*Pain*	Canada	Pain research from 1975 to 2007: A categorical and bibliometric meta-trend analysis of every Research Paper published in the journal, *Pain*	n.r.	n.r.	1975–2007	Journal, *Pain*	0
(34)	2009	Robert et al.	*Scientometrics*	France	Analysis of the medical and biological pain research literature in the European Union: A 2006 snapshot	n.r.	n.r.	2006	CC Life Sciences/Clinical Medicine (ISI-Thompson Scientific)	0
(35)	2010	Robert et al.	*Pain Med*	France	Evolution of the Scientific Literature on Pain from 1976 to 2007	n.r.	n.r.	1976–2007	WoS: Sci-E	0
(36)	2011	Kumar	*J Palliat Care*	India	Reporting Characteristics of Cancer Pain: A Systematic Review and Quantitative Analysis of Research Publications in Palliative Care Journals	Cancer pain	Palliative care	2009–2010	19 palliative/hospice/supportive/end-of-life care journals, Medline CINAHL	0
(37)	2011	Sapunar et al.	*Period Biol*	Croatia	Pain research in Croatia: Analysis of bibliometric trends	n.r.	n.r.	1998–2011	CC Life Sciences/Clinical Medicine/Social and Behavioral Sciences (ISI-Thompson Scientific)	0
(38)	2012	Gupta et al.	*J Indian Acad Oral Med Radiol*	India	Research on orofacial pain in India: A Bibliometric Study	Orofacial pain	n.r.	2006–2010	PubMed, IndMed, PakMediNet	0
(39)	2012	Kissin and Gelman	*J Pain Res*	USA	Chronic postsurgical pain: still a neglected topic?	Chronic postsurgical pain	Specific surgeries	1981–2010	PubMed + four textbooks + manual search + reviewer's refinement	0
(40)	2012	Li et al.	*J Anesth*	China	Citation classics in main pain research journals	n.r.	n.r.	1970–2010	11 specialized pain journals and 22 anesthetic journals	0
(41)	2012	Tolosa-Guzman et al.	*Rev Cienc Salud*	Colombia	Predicción clínica del dolor lumbar inespecífico ocupacional	Non-specific occupational low back pain	n.r.	1985–2012	PubMed, Science Direct, Springer, EBSCO	0
(42)	2013	Balcombe et al.	*J Appl Anim Welfare Sci*	USA	Prolonged Pain Research in Mice: Trends in Reference to the 3Rs	Prolonged pain (neuropathic, inflammatory, chronic)	Mice	1970–2010	PubMed	0
(43)	2013	Kissin	*J Pain Res*	USA	Long-term opioid treatment of chronic nonmalignant pain: unproven efficacy and neglected safety?	Chronic nonmalignant pain	Opioid treatment	1983–2012	PubMed + manual search in the reference lists of reports and reviews	0
(44)	2013	Kumar et al.	*Saudi J Health Sci*	India	Reporting characteristics of cancer pain: A systematic review and quantitative analysis of articles published in cancer journals	Cancer pain	n.r.	2009–2010	PubMed	0
(45)	2013	Leão et al.	*Rev Dor Sao Paulo*	Brazil	Pain research: bibliometric analysis of scientific publications of a Brazilian Research Institution	n.r.	n.r.	2008–2011	PubMed, WoS, Scopus, SciELO, LILACS	0
(46)	2013	Zhang et al.	*Zhonghua Nei Ke Za Zhi*	China	A statistical analysis and perspective of headache-related papers covered in 2011 *PubMed*	Headache	n.r.	2011	PubMed	?
(47)	2014	Chuang and Ho	*Pain Medicine*	Taiwan	A Bibliometric Analysis on Top-Cited Articles in Pain Research	n.r.	n.r.	1900–2011	WoS: Sci-E	0
(48)	2014	Correll et al.	*J Pain Res*	USA	No evidence of real progress in treatment of acute pain, 1993–2012: scientometric analysis	Acute pain	n.r.	1993–2012	PubMed	0
(49)	2014	Kissin	*J Pain Res*	USA	Scientometric assessment of drugs for chronic pain, 1979–2013: rapid growth of publications, paucity of successful drugs	Chronic pain	n.r.	1979–2013	PubMed	0
(50)	2014	Manoharan et al.	*Eur Acad Res*	India	Measuring Specialization of Authors Using Kumaravel's Prepotency Index—A case study of Fibromyalgia	Fibromyalgia	n.r.	1946–2011	PubMed	0
(51)	2014	Onyeka and Chukwuneke	*J Anesth*	Nigeria	Pain research in Africa: a ten-year bibliometric survey	n.r.	n.r.	2002–2012	PubMed, OVID, *African Journal Online* + search with Google Scholar and Dogpile	0
(52)	2014	Vincente-Herrero et al.	*Trauma Fund MAPFRE*	Spain	Low Back Pain and Intervertebral Disk Displacement. Bibliometric and bibliographic review	Low back pain	n.r.	2009–2011	PubMed	0
(53)	2015	Kissin	*Drug Des Dev* and *Ther*	USA	Scientometrics of drug discovery efforts: pain-related molecular targets	n.r.	Drug discovery	1984–2013	PubMed, US patent and trademark office	0
(54)	2016	Caes et al.	*Pain*	Canada	A comprehensive categorical and bibliometric analysis of published research articles on pediatric pain from 1975 to 2010	n.r.	Pediatrics	1975–2010	WoS	0
(55)	2016	Carbone and Austin	*PLOS One*	USA	Pain and Laboratory Animals: Publication Practices for Better Data Reproducibility and Better Animal Welfare	n.r.	Pain in animals	Pre 2011 and 2014–2015	PubMed	0
(56)	2016	Castellini et al.	*Physiotherapy Canada*	Italy	Mechanical Low Back Pain: Secular Trend and Intervention Topics of Randomized Controlled Trials	Low back pain	Clinical Trials	1961–2010	Cochrane Database of systematic reviews	0
(57)	2016	Cuoghi et al.	*J Comtemp Dent Pract*	Brazil	Pain and Tissue Damage in Response to Orthodontic Tooth Movement: Are They Correlated?	Orofacial pain	Orthodontic Tooth movement	undetermined	Scholar Google, CAPES portal of journals	0
(58)	2016	Fan et al.	*Int J Clin Exp Med*	China	Top 100 most-cited articles in low back pain: a 22-year survey of publication activity	Low back pain	n.r.	1994–2015	WoS: Sci-E	0
(59)	2016	Guo et al.	*Lecture Notes in Electrical Engineering*	China	Trend Analysis of Low Back Pain in Ergonomic Area	Low back pain	Ergonomic area	1970–2015	WoS: CC	1
(60)	2016	Gupta et al.	*J Young Pharm*	India	Scientometric Assessment of India's Migraine Research Publications during 2006–15	Migraine	n.r.	2006–2015	Scopus	0
(61)	2016	Huang et al.	*Spine*	China	Top 100 Cited Articles on Back Pain Research	Back pain	n.r.	undetermined	WoS: CC	0
(62)	2016	Ran and Yu	*Chineese J Pain Med*	China	A statistical analysis and perspective of articles related to pain published in *PubMed* from2013 to 2015	n.r.	n.r.	2013–2015	PubMed	0
(63)	2016	Sweileh et al.	*Springer Plus*	Palestine	Worldwide research productivity on tramadol: a bibliometric analysis	n.r.	n.r.	1978–2013	Scopus	0
(64)	2017	Correll and Kissin	*J Anesth Hist*	USA	Publication-Based Academic Interest in Drugs and Techniques for Treatment of Postoperative Pain, 1975–2015	Postoperative pain	Drugs and their administration techniques	1975–2015	PubMed	0
(65)	2017	Franco et al.	*Cienc Cuid Saude*	Brazil	Strategies for pain assessment in critically ill patients: a bibliometric study	n.r.	Critical ill patients	2011–2016	BDEnf, LILACS, Medline, PubMed	0
(66)	2017	Garcia-Rivero	*Rev Cubana Reumatologia*	Cuba	Latin American science of fibromyalgia	Fibromyalgia	n.r.	Undetermined (1994–2016?)	PubMed	0
(67)	2017	Liang et al.	*J Pain Res*	China	Study of acupuncture for low back pain in recent 20 years: a bibliometric analysis *via* CiteSpace	Low back pain	Acupuncture	1997–2016	WoS: Sci-E	1
(68)	2017	Park et al	*Clin Neurol Neurosurg*	South Korea	Top-100 cited articles on headache disorders: A bibliometric analysis	Headache	n.r.	1950–2014	WoS: SCI-E: 3 disciplines (Clinical Neurology/Neuroscience/Medicine, General and Internal)	0
(69)	2017	Robert et al.	*Cephalalgia*	France	Growth of Headache Research: A 1983–2014 bibliometric study	Headache	n.r.	1983–2014	WoS: Sci-E	0
(70)	2018	Damar et al.	*Rev Lat-Amer Enferm*	Turkey	Scientometric overview of nursing research on pain management	n.r.	Nursing issues	1975–2017	WoS	1
(71)	2018	Flores-Fernandez et al.	*Rev Soc Esp Dolor*	Chile	Bibliometric analysis of the *Journal of the Spanish Pain Society*: 2007–2016	n.r.	n.r.	2007–2016	*Journal of the Spanish Pain Society*	0
(72)	2018	Fornaris-Cedeno et al.	*J Oral Res*	Cuba	Trigeminal neuralgia: bibliometric analysis of the fifty top-cited articles in the period 2000–2016	Trigeminal neuralgia	n.r.	2000–2016	Google Scholar	1
(73)	2018	Mendonça and Castro-Lopes	*Scand J Pain*	Portugal	Impact of economic the crisis on pain research: a bibliometric analysis of pain research publications from Ireland, Greece, and Portugal between 1997 and 2017	n.r.	n.r.	1997–2017	WoS	0
(74)	2018	Wang and Zhao	*Medicine*	China	Worldwide research productivity in the field of back pain. A bibliometric analysis	Back pain	n.r.	1995–2016	WoS	0
(75)	2018	Ye et al.	*J Headache* and *Pain*	China	The publication trend of neuropathic pain in the world and China: a 20–years bibliometric analysis	Neuropathic pain	n.r.	1998–2017	WoS: Sci-E, SSci-E, A&HCI, PubMed	0
(76)	2019	Atci	*Ro J Rheumatol*	Turkey	Top 50 cited articles on fibromyalgia: A bibliometric analysis	Fibromyalgia	n.r.	undetermined	WoS: SCI-E, PubMed, Google scholar,	0
(77)	2019	Correll and Kissin	*J Anesth Hist*	USA	Problems with Developments of Breakthrough Analgesics: Recent History *via* Scientometric Analysis	n.r.	13 topics related to analgesia	1982–2016	PubMed	0
(78)	2019	Lei et al.	*BioMed Research Int*	China	A Bibliometric Analysis of Publications on Oxycodone from 1998 to 2017	n.r.	n.r.	1998–2017	PubMed, WoS: SCI-E, SSCI-E, A&HCI	1
(79)	2019	Wang et al.	*Neural Plasticity*	China	Bibliometric Study of the Comorbidity of Pain and Depression Research	n.r.	Depression	1980–2018	WoS: Sci-E	1
(80)	2019	Zheng and Wang	*Med Sci Monit*	China	Publications on the Association Between Cognitive Function and Pain from 2000 to 2018: A Bibliometric Analysis Using CiteSpace	n.r.	Cognitive function	2000–2018	WoS: Sci-E	1
(81)	2020	Anand et al.	*Paediatric* and *Neonatal Pain*	USA	Historical roots of pain management in infants: A bibliometric analysis using reference publication year spectroscopy	n.r.	Pediatrics	1950–2019	WoS: SCI-E, SSCI, A&HCI, CPCI-S, CPCI-SSH, BKCI-S, BKCI-SSH, ESCI, CCR-E, IC	1
(82)	2020	Chen and Wang	*J Pain Res*	China	Bibliometric Analysis of Exercise and Neuropathic Pain Research	Neuropathic pain	Exercise	2005–2019	WoS: SCI-E	1
(83)	2020	Correll and Kissin	*J Anesth Hist*	USA	Academic Interest in Pain: Comparison of Four Specialties with Long-Standing Involvement in Pain Medicine	n.r.	Pain medicine	1998–2017	PubMed	0
(84)	2020	Du et al.	*Front Psychol*	China	The 100 Top-Cited Studies About Pain and Depression	n.r.	Depression	1945–2019	WoS: CC	0
(85)	2020	Lee et al.	*J Pain Res*	South Korea	Bibliometric Analysis of Research Assessing the Use of Acupuncture for Pain Treatment Over the Past 20 Years	n.r.	Acupuncture	2000–2019	WoS	1

n.r., not restricted; SVT, Software Visualization Tool; 202X, undetermined.

**Table 3:** (Group A) Selected variables of 143 documents (75.7%)—***general or classical*** bibliometric analysis of the pain (B/P) literature.

Ref.	Year of publication	Authors	Journal/Source of publication	Country affiliation of first author	Title of the study	Type of Pain studied (as indicated by the authors)	Medical/Scientific context of study	Years covered	Database (s) searched	No. SVTs used
(86)	2020	Orhurhu et al.	*Reg Anesth Pain Med*	USA	Factors associated with academic rank among chronic pain medicine faculty in the USA	n.r.	n.r.	2018 minus year of fellowship graduation as surrogate for beginning of faculty member's academic career	98 academic pain medicine fellowship programs compiled from the American Medical Association Fellowship and Residency Electronic Interactive Database Access, Scopus	0
(87)	2020	Rodriguez and Correia	*Res, Soc and Dev*	Brazil	Chronic pain in hypertension in adults: a bibliometric study	Chronic pain	Hypertension	2010–2020	Bank of Theses and Dissertations of the Coordination for the Improvement of Higher Education Personnel (CAPES), Brazilian Digital Library of Theses and Dissertations (BDTD)	0
(88)	2020	Wang et al.	*J Rehab Med*	China	Exercise for low back pain: a bibliometric analysis of global research from 1980 to 2018	Low back pain	Exercise	1980–2018	WoS: SCI-E, SSCI, A&HCI, CPCI-S, CPCI-SSH,	1
(89)	2020	Weng et al.	*Pain Res* and *Manag*	China	A Bibliometric Analysis of Nonspecific Low Back Pain Research	Low back pain	n.r.	2000–2018	WoS: SCI-E, SSCI, A&SCI, ESCI	1
(90)	2020	Zheng et al.	*J Pain Res*	China	The Trend of Labor Analgesia in the World and China: A Bibliometric Analysis of Publications in Recent 30 Years	Labor pain	n.r.	1988–2018	Scopus, PubMed, WoS, China National Knowledge Infrastructure (CNKI)	0
(91)	2021	Bagcier et al	*Ağrı*	Turkey	Top 100 cited articles on fibromyalgia syndrome: A bibliometric and altmetric analyses study	Fibromyalgia	n.r.	1990–2020	WoS	0
(92)	2021	Bai et al.	*Signa Vitae*	China	Mapping theme trends and knowledge structure of labor analgesia: a quantitative, co-word biclustering analysis of data in 2000–2020	Labor pain	n.r.	2000–2020	PubMed	1
(93)	2021	Chen et al.	*Medicine*	China	The global state of research in pain management of osteoarthritis (2000–2019)	n.r.	Osteoarthritis	2000–2019	WoS: CC	1
(94)	2021	Cheng et al.	*Front Psychiat*	China	Research on Psychache in Suicidal Population: A Bibliometric and Visual Analysis of Papers Published During 1994–2020	Psychache	Suicidal population	1994–2020	WoS: CC	1
(95)	2021	Chi et al.	*BMC Complement Med Ther*	China	Eye acupuncture for pain conditions: a scoping review of clinical studies	n.r.	Acupuncture	1970–2019	PubMed, Cochrane Library, CNKI, Sinomed, Wan Fang data, Chinese Scientific Journals Database (VIP)	0
(96)	2021	Dela Vega	*Neurol Sci*	Philippines	Primary headache research output and association with socioeconomic factors in Southeast Asia: a bibliometric analysis	Primary headache	n.r.	origins to 2020	Scopus, PubMed, EMBASE, Cochrane central register of controlled trials, IMSEAR	1
(97)	2021	Gao et al.	*J Pain Res*	China	Research Trends from 2010 to 2020 for Pain Treatment with Acupuncture: A Bibliometric Analysis	n.r.	Acupuncture	2010–2020	WoS: CC	1
(98)	2021	Garcia et al.	*J Clin Med*	Brazil	A Bibliometric Analysis of Published Literature in Postoperative Pain in Elderly Patients in Low- and Middle-Income Countries	Postoperative pain	Elderly	2001–2021	WoS, Scopus	1
(99)	2021	Guo et al.	*Arch Environ* and *Occup Health*	China	A bibliometric analysis of occupational low back pain studies from 2000 to 2020	Low back pain	Working environment	2000–2020	WoS: CC	2
(100)	2021a	Huang et al.	*J Pain Res*	China	Knowledge Mapping of Acupuncture for Cancer Pain: A Scientometric Analysis (2000–2019)	Cancer pain	Acupuncture	2000–2019	WoS: Sci-E	1
(101)	2021b	Huang et al.	*J Pain Res*	China	Bibliometric Analysis of Functional Magnetic Resonance Imaging Studies on Acupuncture Analgesia Over the Past 20 Years	n.r.	Acupuncture	2001–2021	WoS: SCI-E	1
(102)	2021a	Li et al.	*J Pain Res*	China	Publication Trends and Hot Spots in Chronic Postsurgical Pain (CPSP) Research: A 10-Year Bibliometric Analysis	Chronic post surgical pain	n.r.	2011–2020	WoS: CC	1
(103)	2021b	Li et al.	*Front Human Neurosci*	China	Bibliometric Analysis of Studies on Neuropathic Pain Associated with Depression or Anxiety	Neuropathic pain	Anxiety and depression	2000–2020	WoS	1
(104)	2021	Lu et al.	*Ann Palliat Med*	China	The global trends of migraine research from 2010 to 2019: a scientometric study	Migraine	n.r.	2010–2019	WoS: SCI-E	2
(105)	2021	Luo et al.	*Front Psychol*	China	Study on Pain Catastrophizing From 2010 to 2020: A Bibliometric Analysis *via* CiteSpace	Pain catastrophizing	n.r.	2010–2020	WoS: SCI-E, CCR-E, Index Chemicus	1
(106)	2021	Nosrat et al.	*J Endodont*	USA	Postoperative Pain: An Analysis on Evolution of Research in Half-Century	Postoperative endodontic pain	n.r.	1970–2019	Scopus, PubMed	0
(107)	2021	Özlü	*Int Anatolia Online J*	Turkey	Analysis of publications on myofascial pain syndrome	Myofascial pain	n.r.	1955–2021	Scopus	0
(108)	2021	Pan et al.	*J Pain Res*	China	Knowledge Mapping Analysis of International Research on Acupuncture for Low Back Pain Using Bibliometrics	Low back pain	Acupuncture	1985–2021	WoS: SCI-E	2
(109)	2021a	Park et al.	*J Pain Res*	South Korea	Bibliometric Analysis of Research Trends on Acupuncture for Neck Pain Treatment Over the Past 20 Years	Neck pain	Acupuncture	2000–2021	WoS	1
(110)	2021b	Park et al.	*J Int Kor Med*	South Korea	Bibliometric Analysis of the Effect of Acupuncture on Cancer Pain in the Last 20 Years	Cancer pain	Acupuncture	2001–2020	WoS	1
(111)	2021	Su et al.	*Neural Plasticity*	China	Global Research on Neuropathic Pain Rehabilitation over the Last 20 Years	Neuropathic pain	Neuropathic pain Rehabilitation	2000–2019	WoS: SCI-E	1
(112)	2021	Wang and Meng	*J Pain Res*	China	Global Research Trends of Herbal Medicine for Pain in Three Decades (1990–2019): A Bibliometric Analysis	n.r.	n.r.	1990–2019	WoS: CC	2
(113)	2021	Wang et al.	*Neural Plasticity*	China	Bibliometric Study of Pain after Spinal Cord Injury	n.r.	Spinal cord injury	1990–2019	WoS: Sci-E	1
(114)	2021	Wu et al.	*J Pain Res*	China	Bibliometric Analysis of Research on the Comorbidity of Cancer and Pain	Cancer pain	Cancer	2010–2019	WoS: Sci-E	1
(115)	2021a	Xiong et al.	*Pain Res Manag*	China	Bibliometric Analysis of Research on the Comorbidity of Pain and Inflammation	n.r.	Inflammation	1981–2019	WoS	1
(116)	2021b	Xiong et al.	*Front Neurol*	China	Top 100 Most-Cited Papers in Neuropathic Pain From 2000 to 2020: A Bibliometric Study	Neuropathic pain	n.r.	2000–2020	WoS: SCI-E	1
(117)	2021a	Xu et al.	*Chin J Rehabil Theory Pract,*	China	Advance in Researches of Low Back Pain: A Bibliometrics and Visualization Study	Low back pain	n.r.	2016–2020	WoS: SCI-E, SSCI, A&HCI, CPCIS, CPCI-SSH, ESCI, CCR-E, IC	1
(118)	2021b	Xu et al.	*World Neurosurg*	China	Bibliometric and Visualized Analysis of Neuropathic Pain Using Web of Science and CiteSpace for the Past 20 Years	Neuropathic pain	n.r.	2001–2020	WoS: CC	1
(119)	2021	Yan et al.	*J Pain Res*	China	Research Relating to Low Back Pain and Physical Activity Reported Over the Period of 2000–2020	Low back pain	Physical activity	2000–2020	WoS: CC	1
(120)	2021a	Zhang et al.	*J Pain Res*	China	Overall Reporting Descriptions of Acupuncture for Chronic Pain in Randomized Controlled Trials in English Journals	Chronic pain	Acupuncture Randomized Controlled Trials	Inception to 2020	PubMed, Embase, Cochrane library	0
(121)	2021b	Zhang et al.	*Herald of Medicine*	China	Literature Analysis of Pharmaceutical Service of Cancer Pain	Cancer pain	Cancer	2000–2018	Sinomed, PubMed	0
(122)	2021c	Zhang et al.	*Ann Palliat Med*	China	The 100 top-cited studies on postoperative hyperalgesia in the last 30 years: a bibliometric analysis	Postoperative hyperalgesia	n.r.	1980–2020	WoS	0
(123)	2021a	Zhao et al.	*J Pain Res*	China	A Bibliometric Analysis of Research Trends of Acupuncture Therapy in the Treatment of Migraine from 2000 to 2020	Migraine	Acupuncture	2000–2020	WoS: SCI-E, SSCI, A&HCI, CPCI-S, CPCI-SSH, BKCI-S, BKCI-SSH, ESCI	2
(124)	2021b	Zhao et al.	*J Pain Res*	China	Scientific Knowledge Graph of Acupuncture for Migraine: A Bibliometric Analysis from 2000 to 2019	Migraine	Acupuncture	2000–2019	WoS: SCI-E	1
(125)	2021c	Zhao et al.	*J Pain Res*	China	Bibliometric Analysis of Research Articles on Pain in the Elderly Published from 2000 to 2019	n.r.	Elderly	2000–2019	WoS: Sci-E	1
(126)	2022	Cascella et al.	*J Pain Symp Manag*	Italy	Bibliometric Network Analysis on Rapid-Onset Opioids for Breakthrough Cancer Pain Treatment	Cancer pain	Opioids for cancer pain	1989–2021	WoS	1
(127)	2022	Castro-Osorio et al.	*Gaceta Mexicana de Oncologia*	Colombia	Pain measurement in pediatric cancer patients: A bibliometric analysis	Cancer pain	Pediatrics	to January 2020	PubMed, Proquest, APA PsycArticles	0
(128)	2022	Cheng et al.	*Pain Res Manag*	China	Bibliometric and Visualized Analyses of Research Studies on Different Analgesics in the Treatment of Orthopedic Postoperative Pain	Postoperative pain	Orthopedics	1992–2021	WoS: SCI-E	2
(129)	2022	Di et al.	*Int Urogynecol J*	China	A bibliometric analysis of top-cited journal articles in interstitial cystitis and bladder pain syndrome	Bladder pain syndrome	n.r.	1900–2022	WoS	0
(130)	2022	Du et al.	*Front Mol Neurosci*	China	The Last Decade Publications on Diabetic Peripheral Neuropathic Pain: A Bibliometric Analysis	Diabetic peripheral neuropathic pain	Diabete	2011–2021	WoS: SCI-E	1
(131)	2022	Fan et al.	*Front Neurol*	China	A bibliometric analysis and visualization of tension-type headache	Tension-type headache	n.r.	2022–2021	WoS	2
(132)	2022	Huang et al.	*Front Public Health*	China	International Publication Trends in Low Back Pain Research: A Bibliometric and Visualization Analysis	Low back pain	n.r.	2000–2021	WoS	3
(133)	2022	He et al.	*J Pain Res*	China	A Bibliometric of Trends on Acupuncture Research About Migraine: Quantitative and Qualitative Analyses	Migraine	Acupuncture	1965–2021	Scopus, WoS: CC	1
(134)	2022	Jie et al.	*Chineese J Tissue Eng Res*	China	Visual analysis of patellofemoral pain syndrome research hotspots and content	Patellofemoral pain syndrome	n.r.	2009–2021	WoS, CNKI	1
(135)	2022	Kan et al.	*J Pain Res*	China	Stem Cell Therapy for Neuropathic Pain: A Bibliometric and Visual Analysis	Neuropathic pain	n.r.	1995–2021	WoS	1
(136)	2022	Kissin	*Curr Rev Clin Exp Pharmacol*	USA	Progress in Analgesic Development: How to Assess its Real Merits?	n.r.	Analgesic evaluation	1982–2016	PubMed	0
(137)	2022a	Li et al.	*J Pain Res*	China	Scientific Knowledge Graph and Trend Analysis of Central Sensitization: A Bibliometric Analysis	Central sensitization	n.r.	1998–2020	WoS: SCI-E	1
(138)	2022b	Li et al.	*Front Mol Neurosci*	China	Research Hotspots and Frontiers in Post Stroke Pain: A Bibliometric Analysis Study	Post-stroke pain	n.r.	2010–2021	WoS: CC	1
(139)	2022	Li et al.	*J Pain Res*	China	Knowledge Mapping of Acupuncture for Fibromyalgia from 1990 to 2022: A Bibliometric Analysis	Fibromyalgia	Acupuncture	1990–2022	WoS	2
(140)	2022	Li et al.	*Front Human Neurosci*	China	Research Hotspots and Effectiveness of Transcranial Magnetic Stimulation in Pain: A Bibliometric Analysis	n.r.	Transcranial Magnetic Stimulation	2010–2021	WoS	1
(141)	2022	Liu et al.	*Ann Palliat Med*	China	Knowledge domain and emerging trends in chronic prostatitis/chronic pelvic pain syndrome from 1970 to 2020: a scientometric analysis based on VOSviewer and CiteSpace	Chronic pelvic pain	n.r.	1970–2020	WoS: SCI-E	2
(142)	2022	Liu et al.	*J Pain Res*	China	The Application of Acupuncture Therapy for Postoperative Pain Over the Past 20 Years: A Bibliometric Analysis	Postoperative pain	Acupuncture	2001–2022	WoS	1
(143)	2022	Öntürk Akyüz and Özlü	*Chron Precis Med Res*	Turkey	Analysis of Publications on Pain in The Field of Nursing by Bibliometric Analysis Method	n.r.	Nursing	1942–2021	Scopus	0
(144)	2022	Ortega-Martin et al.	*Rehabilitación*	Spain	Análisis bibliométrico de la evolución temática en fibromialgia y biomecánica (1985–2021)	Fibromyalgia	Biomechanic approach	1985–2021	WoS	1
(145)	2022	Sharma et al.	*Neuroethics*	Canada	Identifying the Presence of Ethics Concepts in Chronic Pain Research: A Scoping Review of Neuroscience Journals	Chronic pain	Ethics	1999–2021	Scholar Google, PubMed	0
(146)	2022	Sung et al.	*Toxins*	South Korea	Clinical Studies of Bee Venom Acupuncture for Lower Back Pain in the Korean Literature	Low back pain	Acupuncture	1999–2022	6 Korean databases	0
(147)	2022	Tafrehi et al.	*J Eval Clin Pract*	Canada	Shifting interpretations in evidence and guidance in pain and opioids research: A bibliometric analysis of a highly cited case series from 1986	Non-malignant pain	n.r.	1986–2020	WoS	0
(148)	2022	Vittori et al.	*Children*	Italy	VOSviewer-Based Bibliometric Network Analysis for Evaluating Research on Juvenile Primary Fibromyalgia Syndrome (JPFS)	Fibromyalgia	Pediatrics	1985–2022	WoS	1
(149)	2022	Weng et al.	*J Headache* and *Pain*	China	Bibliometric study of the global scientific research on pain and disability	n.r.	Disability	1980–2019	WoS: SCI-E, SSCI	1
(150)	2022	Xia et al.	*J Pain Res*	China	Research Trends of Moxibustion Therapy for Pain Treatment Over the Past Decade: A Bibliometric Analysis	n.r.	Moxibustion	2012–2021	WoS	1
(151)	2022	Yang et al.	*J Pain Res*	China	Worldwide Productivity and Research Trend of Publications Concerning Cancer-Related Neuropathic Pain: A Bibliometric Study	Cancer Neuropathic pain	Cancer	2012–2021	WoS	2
(152)	2022	Zhang et al.	*Evidence-Based Comp Altern Med*	China	Effective Oriental Magic for Analgesia: Acupuncture	n.r.	Acupuncture	2011–2021	WoS	1
(153)	2022	Zhu et al.	*Front Neurol*	China	Global Trends and Hotspots in Trigeminal Neuralgia Research From 2001 to 2021: A Bibliometric Analysis	Trigeminal neuralgia	n.r.	2001–2021	WoS: CC	3
(154)	202X	Aguado et al.	*Undetermined*	Mexico	Opioids for pain treatment of Cancer: A Knowledge Maturity Mapping	Cancer pain	Cancer	1985–2020	WoS	0
(155)	202X	Ng and Fakuade	*Undetermined*	Canada	Global Research Trends at the Intersection of Acupuncture and Headache Disorders: A Bibliometric Analysis	Headache	Acupuncture	1965–2021	Scopus	0
(156)	202X	Zhao and Li	*Undetermined*	China	Knowledge Mapping of Migraine: A Bibliometric Analysis (2000–2019)	Migraine	n.r.	2000–2019	WoS: Sci-E	1

n.r., not restricted; SVT, Software Visualization Tool; 202X, undetermined.

**Table 4:** (Group B) Selected variables of 32 documents (18.0%)—***non-specific*** bibliometric analysis of the pain (B/P) literature highlighting pitfalls in publishing.

Ref.	Year of publication	Authors	Journals/Source of publication	Country affiliation of first author	Title of the study	Type of Pain studied (as indicated by the authors)	Medical/Scientific context of study	Years covered	Database (s) searched
(157)	2001	Alvarez-Lario et al.	Ann Rheum Dis	Spain	Acceptance of the diVerent denominations for reflex sympathetic dystrophy	Complex regional pain syndrome	n.r.	1995–1999	(Medline) PubMed
(158)	2004	Von Elm et al.	JAMA	Switzerland	Different Patterns of Duplicate Publication. An Analysis of Articles Used in Systematic Reviews	n.r.	n.r.	1989–2002	A List of systematic reviews in anesthesia, analgesia and critical care. Available at: http://www.hcuge.ch/anesthesie/anglais/evidence/arevusyst.htm. Accessed August 15, 2002
(159)	2009	Ramsdell et al.	Anesth. Analg.	USA	Subspecialty Impact Factors: The Contribution of Pediatric Anesthesia and Pain Articles	n.r.	Perioperative pain	1998, 1999, 2003, 2004	*Anesthesiology, British Journal of Anaesthesia, Anesthesia* and *Analgesia, Canadian Journal of Anesthesia, Pediatric Anesthesia*
(160)	2009	Reeves	Anesth. Analg.	Australia	Increase in Quality, but Not Quantity, of Clinical Trials in Acute Pain: 1992 Versus 2007	Acute pain	Clinical Trials	1992 and 2007	*Anaesthesia, Anesthesiology, Anesthesia* and *Analgesia, British Journal of Anaesthesia, Clinical Journal of Pain, Pain*
(161)	2010	Yim et al.	Korean J Pain	South Korea	Analysis of Statistical Methods and Errors in the Articles Published in the *Korean Journal of Pain*	n.r.	n.r.	2004–2008	*Korean Journal of Pain*
(162)	2012	De Oliveira	Rev Dor Sao Paulo	Brazil	Publication rate of scientific papers presented during the 9th Brazilian Congress on Pain	n.r.	n.r.	2012	Annals of the 9th Brazilian Congress on Pain
(163)	2013	Todorova et al.	Med Princ and Practice	Bulgaria	Complex Regional Pain Syndrome Acceptance and the Alternative Denominations in the Medical Literature	Complexe regional pain syndrome	n.r.	2001–2012	Scopus, PubMed
(164)	2014	Lin and Li	J Tradit Chin Med	China	Comparative literature study between investigations in foreign Science Citation Index journals and Chinese core domestic journals in the treatment of low back pain with acupuncture	Low back pain	Acupuncture	2002–2012	WoS: SCI-E and *four Chinese core domestic journals on acupuncture*
(165)	2015	Gewandter et al.	J Pain	USA	Data Interpretation in Analgesic Clinical Trials with Statistically Nonsignificant Primary Analyses: An ACTTION Systematic Review	n.r.	RCT	2006–2013	*Clinical Journal of Pain, European Journal of Pain, Journal of Pain, Journal of Pain and Symptom Management, Pain, and Pain Medicine*
(166)	2017	Li et al.	Acupunct Med	China	Reporting characteristics and risk of bias in randomised controlled trials of acupuncture analgesia published in PubMed-listed journals	n.r.	Acupuncture	undetermined	PubMed
(167)	2017	Minguez et al.	Anesth Analg	Spain	Methodological and Reporting Quality of Systematic Reviews Published in the Highest Ranking Journals in the Field of Pain	n.r.	n.r.	2005–2015	*Anesthesiology, Pain, British Journal of Anaesthesia, Pain Physician, Anesthesia* and *Analgesia, Anaesthesia, and Regional Anesthesia and Pain Medicine*
(168)	2018	Dosenovic et al.	BMC Med Res Method	Croatia	Comparison of methodological quality rating of systematic reviews on neuropathic pain using AMSTAR and R-AMSTAR	Neuropathic pain	RCT	From the earliest date that each database allowed up to 2015	Medlline, Cochrane Database of Systematic Reviews, DARE, CINAHL and PsycINFO
(169)	2018	Piotrowski	J Ind Acad Applied Psychol	USA	MMPI-Related Pain Literature: Identifying Neglected Research Domains	n.r.	n.r.	1992–2017	PsycINFO
(170)	2018	Piotrowski	North Am J Psychol	USA	MMPI-related Pain Research through the Lens of Bibliometric Analysis: Mapping Investigatory Domain	n.r.	n.r.	1992–2017	PsycINFO
(171)	2018	Szilagyi and Bornemann-Cimenti	Pain Med	Austria	Gender Distribution of Authorship in Pain Publications Is More Balanced than in Other Scientific Fields	n.r.	n.r.	2005 and 2015	WoS: SCI-E, SSCI, A&HCI, ESCI
(172)	2019	Akkoc	J Int Med Res	Turkey	Publication rates of abstracts presented at the World Congress on Pain held by the International Association for the Study of Pain in 2010	n.r.	n.r.	2010	Congress booklet of the 2010's World Congress on Pain
(173)	2019	Nascimento et al.	Arch Phys Med Rehab	Brazil	Abstracts of Low Back Pain Trials Are Poorly Reported, Contain Spin of Information, and Are Inconsistent with the Full Text: An Overview Study	Low back pain	n.r.	2010–2015	Physiotherapy Evidence Database (PEDro)
(174)	2019	Piotrowski	SIS J. Proj. Psy. and Ment. Health	USA	Fibromyalgia, Low Back Pain, Osteoarthritis, Myofascial Pain, and Complex Regional Pain Syndrome: Predominant Assessment Measures in Research	Fibromyalgia, Low back pain, Osteoarthritis, myofacial pain, and CRPS	n.r.	undetermined	PsycINFO
(175)	2019	Saric et al.	Eur J Pain	Croatia	Comparison of conference abstracts and full-text publications of randomized controlled trials presented at four consecutive World Congresses of Pain: Reporting quality and agreement of results	n.r.	n.r.	2008–2010–2012–2014	Congress booklets of the 2008,2010,2012, and 2014's World Congress on Pain
(176)	2020	Karri et al.	Reg Anesth Pain Med	USA	Exploration of Gender-Specific Authorship Disparities in the Pain Medicine Literature	n.r.	n.r.	2014–2018	*Pain, Pain Practice, The Journal of Pain, Pain Physician, Neuromodulation, Pain Medicine, Reg Anesth Pain Med*
(177)	2020a	Nascimento et al.	Eur Spine J	Brazil	Journal impact factor is associated with PRISMA endorsement, but not with the methodological quality of low back pain systematic reviews: a methodological review	Low back pain	n.r.	2015–2017	Physiotherapy Evidence Database (PEDro)
(178)	2020b	Nascimento et al.	J Orthoped and Sport Phys Ther	Brazil	Eight in Every 10 Abstracts of Low Back Pain Systematic Reviews Presented Spin and Inconsistencies with the Full Text: An Analysis of 66 Systematic Reviews	Low back pain	n.r.	2015–2017	Physiotherapy Evidence Database (PEDro)
(179)	2020	Piotrowski	SIS J. Proj. Psy. and Ment. Health	USA	Pain Assessment: Most Prominent Measures/Tests in the Research Literature, 2006–2018	n.r.	n.r.	2006–2018	Textbooks, PsycINFO, ProQuest
(180)	2020	Saric et al.	J Clin Epidem.	Croatia	Conference abstracts describing systematic reviews on pain were selectively published, not reliable, and poorly reported	n.r.	n.r.	2008–2010–2012–2014–2016	Congress booklet of the 2008,2010,2012,2014, and 2016's World Congress on Pain
(181)	2021	Mukhdomi et al.	Pain Practice	USA	An evaluation of impact factor bias of clinical trials published in pain journals	n.r.	Clinical trial	2012–2018	*Pain, J Pain, Eur J Pain, Reg Anesth Pain Med, Clin J Pain, Pain Physician, Pain Med, Pain Pract, Pain Res Manag*
(182)	2021	Nascimento et al.	Braz J Phys Ther	Brazil	Factors associated with the reporting quality of low back pain systematic review abstracts in physical therapy: a methodological study	Low back pain	n.r.	2015–2017	Physiotherapy Evidence Database (PEDro)
(183)	2021	Thompson et al.	Pain Manag	USA	Evaluation of “spin” in the abstracts and articles of randomized controlled trials in pain literature and general anesthesia	n.r.	Clinical trial	2016–2017	*Brit J Anaesth, Anesthesiol, Anesth* and *Analg, Anaesthesia, Reg Anesth Pain Med, Pain, J Pain, Pain Phys, J Pain Symp Manag, Pain Med*
(184)	2021	Zhang and Cheng	Headache	USA	Interdisciplinary influences in headache literature: A network citation analysis of PubMed Central articles	Headache	n.r.	1826–2019	PubMed
(185)	2022	Lisicki et al.	Cephalalgia	Argentina	Bridging the gaps of headache care for underserved populations: Current status of the headache field in Latin America	Headache	n.r.	2010–2020	PubMed
(186)	2022	Munro et al.	Vet Anaesth Analg	Canada	Randomization, blinding, data handling and sample size estimation in papers published in Veterinary Anaesthesia and Analgesia in 2009 and 2019	n.r.	Animal	2008–2009–2018–2019	*Veterinary Anaesthesia and Analgesia*
(187)	2022	Schwartz et al.	Pain	USA	Pharmacologic therapies for neuropathic pain: an assessment of reporting biases in randomized controlled trials	Neuropathic pain	Clinical trials	undetermined	PubMed, RReACT, Cochrane Central Register of Controlled Trials
(188)	2022	Szilagyi et al.	Scientometrics	Austria	Citation of retracted research: a case-controlled, ten-year follow-up scientometric analysis of Scott S. Reuben's malpractice	n.r.	n.r.	2009–2019	WoS, PubMed, Google scholar

n.r., not restricted.

**Table 5:** (Group C) Selected variables of 14 documents (c. 8%)—***miscellaneous*** bibliometric analysis of the pain (B/P) literature addressing objectives not in Groups A or B.

Ref.	Year of publication	Authors	Journal/Source of publication	Country affiliation of first author	Title of the study	Type of Pain studied (as indicated by the authors)	Medical/Scientific context of study	Years covered	Database (s) searched
(189)	2011	Kissin	Scientometrics	USA	Can a bibliometric indicator predict the success of an analgesic?	n.r.	Drug discovery	Undetermined	All biomedical journals covered by Medline
(190)	2016	Wen et al.	Zhonghua Nan Ke Xue	China	International focuses in the studies of chronic pelvic pain syndrome: A social network analysis	Chronic pelvic pain syndrome	n.r.	Until 2015	PubMed
(191)	2018	Araujo et al.	J Med Internet Res	Brazil	Impact of Low Back Pain Clinical Trials Measured by the Altmetric Score: Cross-Sectional Study	Low back pain	Clinical trials	2010–2015	Physiotherapy Evidence Database (PEDro)
(192)	2018	Liu et al.	Chin Med J	China	Multimodal Magnetic Resonance Imaging Studies of Migraines Related to Increasing Risk Factors for Brain Lesions Would Be an Optimal Research Focus: A Pilot Literature Citation Analysis	Migraine	n.r.	All years	WoS: SCI-E, CPCI-S, CCR-E, Index Medicus
(193)	2019	Contiero et al.	J Agricult and Environ Ethics	Italy	Pain in Pig Production: Text Mining Analysis of the Scientifc Literature	n.r.	Pain in pig	1970–2017	Scopus
(194)	2019	Oh et al.	J Pain Res	South Korea	Construction And Analysis of The Time-Evolving Pain-Related Brain Network Using Literature Mining	n.r.	n.r.	Beginning of the 1970's to 2015	PubMed
(195)	2019	Wrigley et al.	J Med Lib Assoc	USA	Bibliometric mapping for current and potential collaboration detection	General, and musculoskeleton pain	n.r.	Undetermined	EndNote library of Duke School of Medicine publications
(196)	2020	D’Amico et al.	Int J Environ Res and Public Health	Italy	Mapping Assessments Instruments for Headache Disorders against the ICF Biopsychosocial Model of Health and Disability	Headache	Biopsychosocial environment	2015–2020	Scopus
(197)	2020	Rahimi et al.	J Scientometric Res	Iran	Social Influence, Research Productivity and Performance in the Social Network Co-authorship: A Structural Equation Modelling.	Headache	Social influence	2005–2017	WoS
(198)	2020	Tighe et al.	Pain Med	USA	Forty-two Million Ways to Describe Pain: Topic Modeling of 200,000 PubMed Pain-Related Abstracts Using Natural Language Processing and Deep Learning–Based Text Generation	n.r.	n.r.	1940–2017	PubMed
(199)	2021	Araujo et al.	Braz J Phys Ther	Brazil	The impact of low back pain systematic reviews and clinical practice guidelines measured by the Altmetric score: Cross-Sectional study	Low back pain	n.r.	2015–2017	PEDro
(200)	2021	Fassoulaki et al.	Anesthesia	Greece	Bibliometric analysis of alternative performance metrics for peri-operative, critical care and pain medicine journals	n.r.	Peri-operative, critical care and pain medicine	2018–2019	18 journals
(201)	2021	Fassoulaki et al.	Ind J Anaesth	Greece	Impact of Altmetrics in evaluation of scientific journals, research outputs and scientists’ careers: Views of editors of high impact anaesthesia, critical care and pain medicine journals	n.r.	Anesthesia, critical care ad pain medicine	Undetermined	7 Anesthesia journals, 9 Critical care medicine journals, 11 Pain Medicine journals
(202)	2022	ElHawary et al.	Ann Surg	Canada	Efficacy and Safety of Migraine Surgery	Migraine	Migraine surgery	Undetermined	EMBASE, PubMed

n.r., not restricted.

### 3.1. Datasets

The datasets are comprised of traditional and well-established institutional databases; holdings or collections of specialized libraries; one or several appropriate scientific journals; compilations of Congress or Conference Abstracts; two or more databases with duplicate documents removed; and sometimes a mixture of any (or all) of the above.

As expected, the *Web of Science* (around 47% of all B/P studies), *PubMed* (30%), and *Scopus* (7%) were generally the most used databases, singly or in some combination (Tables 2–5). Other databases (often country-based) were also used, but to a lesser extent.

Supplementing the three major databases, some studies have integrated their own national or specialized databases: for example, *SinoMed* (Chinese), *Cochrane Library* (collection of medical/healthcare databases). The diversity of the databases used reflect the comprehensive approaches developed to search and retrieved publications on various aspects of pain. For example, *PsycINFO* was used to identify methods of pain assessment (i.e., measures, scales, inventories, tests) in the research literature (170, 174); *BDENF*, a Brazilian nursing and thematic database which is part of the Latin American and Caribbean Health Sciences Information System, was used to identify papers related to the diagnosis of pain by nurses (28).

Most of the papers in our study of the B/P literature used only one database; however, several studies used two or more:

(1) *China National Knowledge Infrastructure* (*CNKI*) full-text database was combined with: *WoS* to analyze the patellofemoral pain syndrome hotspots (134); *PubMed*, the *Cochrane Library* and three Chinese databases to describe the literature on eye acupuncture for pain conditions (95); *WoS*, *Scopus*, and *PubMed* to analyze trends in labor analgesia research publications (90).
(2) *SinoMed* and *PubMed* were searched for publications on the pharmaceutical service of cancer pain in China (121).
(3) *African Journal OnLine Database* (AJOL) was combined with *PubMed* and *Ovid* to survey the pain research literature from Africa (51).
(4) *Index Medicus for the South-East Asia Region* (*IMSEAR*), *Scopus*, *PubMed*, *EMBASE*, and the *Cochrane Central Register of Controlled Trials* (*CENTRAL*) were searched for publications on primary headache studies with at least one author affiliated with a South-East Asian country (96),
(5) Six Korean databases (the *Korea Institute of Science and Technology Information*, the *Korean Traditional Knowledge Portal*, *KoreaMed*, *OASIS*, *RISS*) and the *National Library of Korea* were used to identify all of the characteristics of bee venom acupuncture for the treatment of lower back pain in the Korean literature (146).

Other national and/or specialized databases were also searched for bibliometric analysis of the pain literature: *Literatura Latino Americana em Ciências da Saúde (LILACS*) and *Scientific Electronic Library Online (SciELO)* (45, 162); *Indian Medical Journals (IndMED)* and the *Medical Information Gateway of Pakistan* (*PakMediNet*) (38); *PsycLIT* (15); *Hungarian Medical Bibliography* (*HBM*) (23); *PEDro (Physiotherapy Evidence Database)* (173, 177, 178, 182, 191, 199); *Ageline* and *SocioAbs* (18).

A few studies used only journals to form their datasets and even fewer used a single journal with varying time spans: Keefe et al. followed the evolution of papers related to pain in *Pyschosomatic Medicine* for 61 years from 1939 to 1999 (20); Mogil et al. analyzed 33 years of the journal *Pain* from 1975 to 2007 (33); a survey of 10 years of *Revista de la Sociedad Española del Dolo*r by Flores Fernandez et al. provided a descriptive bibliometric analysis of the journal's productivity from 2007 to 2016 (71); an editorial by Turk in 2010 listed the most frequently cited articles for 8 years from 2002 to 2009 in the *Clinical Journal of Pain* (24); and Yim et al. focused on the statistical methods and errors in the articles published over 5 years in the *Korean Journal of Pain* from 2004 to 2008 (161).

Added to these few studies of single-journal datasets, five investigations used a group of journals: an earlier study to compare the Impact Factors (IFs) of pediatric anesthesia and pain articles from four anesthesia journals (159); a second study to provide a comprehensive list of the top-100 classic citations in the specialty of pain research from 11 pain-specific and 22 anesthetic-related journals (40); a third study to compare the IF with the Altmetric IF (quantitative and qualitative measures, complementary and/or supplementary to traditional citation-based metrics) of 18 perioperative, critical care and pain medicine journals (200); a fourth study to evaluate the IF bias of clinical trials published in nine pain journals (181); and a fifth study using ten pain or anesthesiology journals to illustrate the presence of “*spin*” in the abstracts and articles of RCTs (183).

Additionally, Congress or Conference Abstracts have been used as datasets for several investigations (172, 175, 180), and a list of 98 academic pain medicine fellowship programs—compiled from the American Medical Association Fellowship and Residency Electronic Interactive Database Access—was used to examine the influence of research productivity to attain professorships among members of the chronic pain medicine faculty (86).

Whatever the subject of interest may be, the choice of datasets for subjects such as our B/P analysis relies on a combination of the following: desired level of scientific reliability, scope, and pertinency of the dataset; accessibility of the dataset—for free or for a fee; ease with which the retrieved documents can be processed by, for example, data visualization software; and familiarity of the dataset to the researchers.

### 3.2. Document selection

The dataset from which documents (e.g., articles, abstracts, reports) for bibliometric analysis is obtained and in which enough attention is paid to terms and phrases describing pain is key to determining the quality of the selection and the accuracy of the results. The selection process is often greatly simplified when investigators choose publishing outlets that only contain “pain documents”: pain-focused journals, proceedings of congress on pain, list of publications of researchers working in a laboratory/medical center/institution dedicated to pain research. However, this is often not the case, and the strategy then becomes how to extract a set of “pain” documents from a dataset that contains both “pain” and “non-pain” documents. Depending on the aim(s) of the studies, investigators need to define a set of pain keywords, key phrases, and/or criteria, which will most likely retrieve the desired papers. The field of basic and clinical pain research is characterized by multiple terms, often by an overlapping of similar pain phenomena for different pain concepts, and by the frequent evolution of pain terminology leading to the difficulty of selecting an appropriate set of documents. A great variation exists in the choice of pain terms: the pain literature is analyzed without any specificity; a large set of pain terms are used; pain synonyms/analogues/related terms (e.g., nociception, analgesia, neuralgia) are employed (30, 34, 35, 47, 123); the number of pain terms used may exceed 20 (35, 37, 150) and can be up to 30 (54, 79), even though a few studies only use the single word “pain” (80, 100, 112, 140, 143). Alternatively, when a specific pain phenomenon is targeted (e.g., fibromyalgia, headache, or low back pain), the choice of pain-terms is reduced to either one (19, 25, 108, 126, 131, 133, 137, 144) or very few (<5) keywords (29, 68, 69, 73, 96, 155, 185).

If the selection of pain terms plays a major role in the retrieval process, and if the papers give detailed and replicable descriptions of the procedures used, then the results are presumed to be accurate. However, studies often lack precision in the selection process: for example, in the study of Dela Vega and colleagues in 2021 to assess headache research impact and productivity among 11 SEA (Southeast Asian) countries, a “systematic search” was performed that included one or more of four pain-related terms or phrases (primary headache, migraine, trigeminal autonomic cephalalgia, and tension-type headache) whenever at least one author is from a SEA country. However, immediately following the search strategy is the ambiguous sentence: “*Equivalent terms for “migraine”, “tension-type headache,” and “trigeminal autonomic cephalalgia” were also inputted in the search string*” (96). No “equivalent” terms were stated; hence, readers are left to *guess* what additional terms or phrases were included. In other studies, the fields in which the pain keywords are searched (*Title*, *Title/Abstract*, *Title/Abstract/Keywords*) are not indicated (58, 72, 74, 85, 106, 185). This omission can have a dramatic effect on the number of documents retrieved and consequently on the analysis. A quick search on *PubMed* for 2001–2021 inclusive, retrieved 153,365 documents with the term “pain” in only the *Title* field; one using the *Title and Abstract* fields more than tripled the dataset to 545,272 documents.

**Figure 1:**
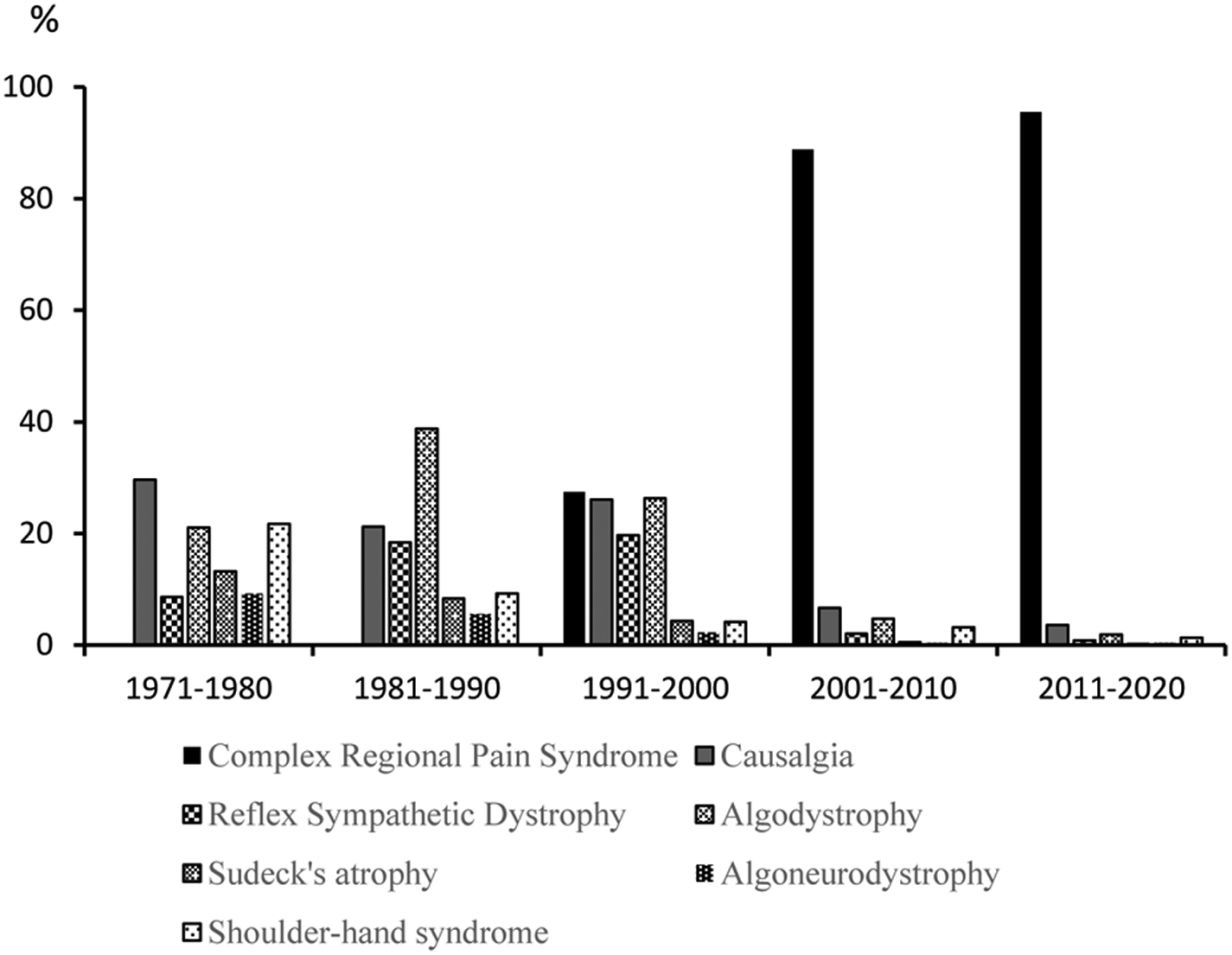
Evolution of the number of complex regional pain syndrome publications in *PubMed* from 1971 to 2020. The seven keywords/keyphrases were searched in the Title/Abstract fields and shown in five 10-year periods (1971–1980, 1981–1990, …), each period totaling 100% of publications.

A further difficulty in identifying the pain-related literature is linked to the intrinsic complexity of pain itself. *Firstly*, the history of the syndrome *causalgia* was initially published by Mitchell and colleagues in 1864 (203). Since then, many synonyms (algodystrophy, algoneurodystrophy, Sudeck's atrophy, Reflex Sympathetic Dystrophy, Complex Regional Pain Syndrome) were used to refer to this syndrome (163, 204, 205), and finally, the phrase Complex Regional Pain Syndrome (CRPS) was proposed by the International Association on Pain in 1994. Hence, a consequence for a bibliometric study is the diffusion of publications focusing on the same phenomenon but appearing with different names; this can lead to errors in estimating the number of publications. A quick search on *PubMed* revealed that from 1971 to 2000, several synonyms (causalgia, algodystrophy, algoneurodystrophy, Reflex Sympathetic Dystrophy, Sudeck's atrophy) were used; these were rapidly replaced from 2000 onwards by the term *Complex Regional Pain Syndrome* (Figure 1). These observations agree with two previous studies (157, 163). Several other pain terminologies were also modified: “carpalgia” has replaced “pain in the wrist” (206); the term “trigeminal neuralgia” has supplanted “tic douloureux”; several recent papers favor the term “Persistent Spinal Pain Syndrome” for CPSS (Chronic Pain after Spinal Surgery) or FBSS (Failed Back Surgery Syndrome) (207–210); and after centuries of using dozens of terms such as “muscular rheumatism”, “muscle calluses”, “chronic rheumatic myitis”, “fibrositis”, “muscular hardening” (211), the term “fibromyalgia” emerged in the mid-1970s (212). Its recognition as a syndrome occurred several years later (213), and the first criteria for the classification of FMS (Fibromyalgia syndrome) in a well-designed, blinded study were published by the American College of Rheumatology in 1990 (214).

*Secondly*, the emergence of new pain terms, due mainly to continuing research and discovery, has added further complications in assembling pain-related literatures for bibliometric studies. An example is the term “nociplastic pain” proposed by Kosek and colleagues in 2016 (215) to describe a “mechanistic descriptor for chronic pain states not characterized by obvious activation of nociceptors or neuropathy … but commonly experienced by people worldwide” (216). There is also the concept of *mixed pain* referring to patients who have a substantial overlap of nociceptive and neuropathic pain symptoms in the same body area (217). Continued interest in *mixed pain* has resulted in the recognition of the term by the IASP/International Association for the Study of Pain (218). Another example is the growing utilization of the term “localized neuropathic pain” that concerns approximately 60% of neuropathic pain patients (219) and is supported by the fact that pain localization is one of the hallmarks when determining the choice of first-line treatment in patients with neuropathic pain (220).

Additionally, the following observations are indicative of a more global, complex, and evolving landscape of the pain literature:

(1) the continuous reconsideration by the scientific community of the IASP definition of *pain* (221–223) as well as the recognition and “acknowledgement of pain as a pathologic entity in its own right” (224);
(2) an IASP Working Group proposal to the International Classification of Diseases (ICD-11) for a classification system of *chronic pain* with the aim of advancing the recognition of *chronic pain* as a health condition term on its own (225);
(3) the multiplicity of diagnostic tools for *neuropathic pain* (226);
(4) the ambiguity of some pain terms (227);
(5) the still unspecified international classification of diseases (ICD) code for *low back pain* (228);
(6) the numerous measures/tests for pain assessment existing in the pain literature—Piotrowski listed 130 measures/tests found in journal articles in the database *ProQuest* (179); and
(7) the cultural influences on pain (229) and the ethnic differences in pain and its management (230).

From these observations, some background knowledge of the field of pain research (e.g., history, terminology) is necessary to engage in a bibliometric investigation and to produce a high-quality study.

### 3.3. Publication environment

Of the 189 B/P papers for which the sources of publication were available and retained in this study were published in 122 different journals (Tables 2–5).

By far, the *Journal of Pain Research* with 29 B/P papers is the most productive; journals with five or fewer B/P papers include: *Pain* with five B/P papers, *Anesthesia* and *Analgesia*, *Pain Medicine*, and *Pain Research and Management* have four B/P papers each, five journals have three B/P papers, 14 journals published two B/P papers, and 100 journals each have one B/P paper. As expected, a large number of papers (67 or 35.4%) in our 189 B/P studies were published in pain-focused journals either with an international audience such as *Pain* (15, 30, 33, 54), *The Journal of Pain* (165), *The Journal of Pain Research* (114, 133), *Pain Management* (183), the *Scandinavian Journal of Pain* (73), etc.; or mostly a national readership such as *Ağrı* (91), the *Chinese Journal of Pain Medicine* (62), *Revista de la Sociedad* *Española de Dolor* (17, 71), the *Korean Journal of Pain* (161), or the *Revista Dor São Paulo* (45, 162).

Beyond this core of pain-focused journals, but still highly related to the basic and clinical aspects of pain research, more than 15 studies have been published in journals indexed in the field of Neurosciences and Neurology: e.g., *Neurological Sciences* (96), *Anesthesia* (200), *Journal of Anesthesia* (51), *Frontiers in Molecular Neuroscience* (138), *Neuroethics* (145), or *Frontiers in Neurology* (153).

Additionally, a large number of studies have been published in journals focusing on a specific field associated with the type of pain studied: e.g., *Gaceta Mexicana de Oncologia* (127) for Cancer; *Paediatric and Neonatal Pain* (81) or *Children* (148) for Pediadrics; *Geriatric Nursing* (18) for Geriatrics; *Archives of Physical Medicine and Rehabilitation* (173) or *Journal of Rehabilitation Medicine* (88) for Rehabilitation; *Rheumatologίa Clίnica* (25) or *Revista Cubana de Reumatologίa* (66) for Rheumatology, *Journal of Psychosomatic Research* (26) or *Frontiers in Psychology* for Psychology and Mental Health (105).

On the other side of the disciplinary spectrum, we note that many B/P papers are published in such diverse fields as: Agriculture with the paper of Contiero et al. published in the *Journal of Agricultural and Environmental Ethics* (193); Veterinary science with the recent study of Munro et al. in *Veterinary Anaesthsia and Analgesia* (186); Information Science with papers in *Scientometrics* (34, 188, 189), and *Journal of Scientometric Research* (197); Health Sciences Librarianship with the Virtual Project authored by Wrigley et al. in the *Journal of the Medical Library Association* (195); General Topics such as pain and laboratory animals by Carbone and Austin in *PLoS One* (55); and the world research productivity on tramadol by Sweileh et al. in *SpringerPlus* (63).

Among the 122 journals publishing B/P papers, the *Journal of Pain Research* is notable: firstly, it has 29 B/P papers while all the other journals contain five or fewer B/P papers (see above); secondly, 23 of the 29 B/P papers were published in the most recent years (2020–2022); and thirdly, 22 (75.9%) papers were authored by Chinese researchers—over twice the rate (36.5%) as the overall number (69 of 189—see Table 1) of Chinese B/P papers. Additionally, in 2021–2022, the *Journal of Pain Research* published three B/P papers on the same topic “migraine/acupuncture” (123, 124, 133). Finally, discussions below in the sub-section “Anomalies …” pertain to many B/P papers in the *Journal of Pain Research* and offer strong incentives for reflection on the possible overlap of future B/P studies.

At the document-type level within journals, most of the B/P papers are “Articles” (Article, Original paper, Original research, Research article) or “Reviews” (Review, Comprehensive review, Topical review, Mini review). However, several B/P papers are presented under other labels such as “Meta-analysis” (60, 93, 167), “Short communications” (26), “Letters” (14), “Virtual project” (195), “Correspondence” (192), or “Editorial” (24).

This diversity in the publishing format is not surprising and illustrate the variety of approaches followed to investigate the pain literature through a bibliometric prism. It can be viewed as a positive contributing factor to heighten the visibility of pain research among the scientific and medical community.

### 3.4. Journal impact factor (JIF) or impact factor (IF)

Within the following B/P papers, the JIF is employed as a bibliometric index; three sets are discussed, each showing how the JIF is used:

(1) The first set includes bibliometric studies in which the IF appears as a “journal-level bibliometric index”: generally, the authors provide tables in their Results section that include listings of journals containing “pain papers” in decreasing frequency order accompanied by the JIF of each journal (30, 31, 34, 35, 40, 45, 61, 63, 67, 69, 72, 79, 80, 82, 97, 104, 111–115, 123, 130–132, 135, 138, 139, 141, 150, 151). Within these 31 B/P papers, the JIF is only one of several indexes (e.g., numbers of papers, numbers of citations, h-index) used in their analyses. Added to these studies are the papers where the JIF is used to analyze the publishing behavior of B/P papers in journals with “high” JIFs vs. other journals (40).
(2) The second set includes B/P papers in which the JIF is used in either the methods and/or the discussion/analysis sections:
  a. Gomez and Conlee aimed at analyzing the journal requirements for authors for inclusion of detailed information on animal pain and distress—a large part of the journals in their databases are selected based on their high impact factors (27);
  b. Lin and Li selected “*four Chinese core domestic journals on acupuncture that had the highest impact factors amongst other Chinese journals on acupuncture*” to compare Chinese articles on the treatment of low back pain using acupuncture with similar non-Chinese articles published in journals of the *Science Citation Index* (164);
  c. Minguez et al. analyzed the methodological and reporting quality of pain systematic reviews contained in seven top-ranking (with high IFs) journals based on the *Journal Citation Reports* category, *Anesthesiology* (167);
  d. Reeves compared the increase in quality but not in quantity of clinical trials papers on acute postoperative pain published in six anesthesiology journals with the highest Impact Factors (160);
  e. Terajima and Aneman compared the topmost cited anesthesia/pain papers published in anesthesia and pain journals *versus* the topmost cited anesthesia/pain papers published in other leading general medical journals with high IFs (22);
  f. Karri et al. evaluated the gender-specific authorship disparities in high versus low impact journals (176); and
  g. Kissin and Gelman assessed the publications on chronic postsurgical pain in leading journals (39), while Kissin assessed the long-term opioid treatment of chronic nonmalignant pain (43) and evaluated drugs for chronic pain (49).
(3) The third set includes a few miscellaneous uses of the IF index with various aims, for example:
  a. To evaluate the number and type of Croatian publications in the field of pain research, and to compare it with an identical dataset by researchers from Graz, Austria, as they have similar scientific productivity (37);
  b. to highlight the necessity of developing and increasing pain research in Africa (51);
  c. to compare the impact on the scientific literature (using JIF) with some social media index such as Altmetric scores (alternative metrics complementary to citation-based metrics, see: www.scienceeditorium.com/blog/journal-impact-factor-versus-altmetrics/) (191, 199, 200);
  d. to suggest the use of Altmetric analysis as an alternative to the JIF (91);
  e. to study the publications issued from abstracts presented at the 2010 World Congress on Pain (172);
  f. to analyze whether there is an association of the JIFs publishing low back pain systematic reviews with journals endorsed by recommendations of the Preferred Reporting Items for Systematic Reviews and Meta-Analyses (PRISMA) and the reviews methodological quality (177); and
  g. to see if a difference exists in the citation of retracted articles of a pain researcher between high-impact and low-impact journals (188).

In summary, the criticisms that have accompanied the Impact Factor for decades—mainly directed at its misused and/or misinterpretation (231, 232)—the presence and influence of the IF in bibliometric papers, including those in pain-related fields, will most likely continue. Nevertheless, it remains the responsibility of the scientific and medical communities, both authors and readers, to use and interpret the information provided by this index appropriately.

### 3.5. Citation analysis

During the last decades, author, or paper/article citation counts (as measures of impact or influence) have rapidly been considered one of the main metrics in any bibliometric toolkit.

Among the various B/P studies, a dozen or so were mainly aimed at investigating the pain literature through citation analysis as indicated in their titles (“Top-cited articles in …” or “Most frequently cited papers in …”). Two studies considered all papers in the pain-related literature (16, 47); others were restricted to literatures of specific pain such as fibromyalgia (76, 91), neuropathic pain (116), back pain (58, 61), postoperative hyperalgesia (122), headache (68, 120) trigeminal pain (72); and one study combined the literatures of pain and depression (84).

Although most of the citation studies were similarly arranged; that is, by the distribution of the top-cited papers over journals, countries and/or institutions, some studies were limited to brief descriptions (16, 58, 61, 72, 76, 84), while others were more comprehensive in either discussing their findings (47, 105, 133, 137, 141, 200), integrating their results with Altmetric analysis (91), or using existing methods such as PageRank and HITS (Hyperlink-induced topic search, see http://pi.math.cornell.edu/∼mec/Winter2009/RalucaRemus/Lecture4/lecture4.html) to augment citation analysis in the *PubMed* database (184).

However, though the studies mentioned above carry information about the pain literature, we should remind ourselves just what citation analysis comprises:

• individual citation for which a large array of heterogeneous motivation may influence the choice of researchers in citing one paper rather than another paper (233);
• limitation in choosing parameters which may bias the results, such as: datasets, search terms, document-type(s), time period. The influence of the search criteria is illustrated in a short comparison made between two studies of the “top 100” articles in radio graphics—using different databases with different journals indexed and different time selection criterion—produced an overlap of only 70%; that is, each study identified 30 frequently cited articles unique to the search criteria used (234);
• just one of the many bibliometric indexes available and provides only a partial and non-qualitative view of a literature landscape (235).

In summary, citation analysis is a powerful investigative tool; however, the conclusions reached from studies relying only on this metric must be scrutinized. Readers should always remind themselves that the integration of citation analysis within a wider bibliometric approach is needed to avoid any mis-, under- or over-interpretation.

### 3.6. Gender of first authors

Not surprisingly, the gender theme of authors of the pain literature has either been the focus or largely integrated in several bibliometric studies. At the end of the 20th century, Strassel et al. performed a citation analysis of contributors to the pain and analgesia literature and observed that “*Few women were first authors of any most frequently cited paper*” (16). Two decades later, in a brief commentary, an impressive increase of the percentage of female authors in pain-related publications from 26.4% in 2005 to 40.5% in 2015 was noted by Szilagyi and Bornemann-Cimenti (171). This trend corroborates the results of two literature studies: in neurology by Nguyen et al. (236) and in neuroscience by Dworkin et al. (237). In the first study, the authors noted the increase of female authorship in journals classified in the *MeSH Journal Category* “Pain” from 7.6% (1946–2001) to 35.4% in 2002–2020 (236). Although this positive trend is certainly encouraging and heading towards parity, a recent study revealed a highly skewed gender disparity in the publications of the pain research community with a prevalence of 70.6% male first authors and 81.6% male senior authors (176). Their results, however, should be viewed with caution since:

• only 20 of the highest cited papers, each of seven journals affiliated with the seven leading societies in the academic pain community, were analyzed;
• only papers authored by persons in the USA formed their dataset;
• only the principal (first and last) authors were considered; and finally,
• only a 5-year (2014–2018) span was considered.

Nonetheless, a general trend toward gender authorship parity is evident, even if obstacles remain. Following the evolution of gender disparity in pain literature and comparing it with those in closely related disciplines such as neurology or neurosciences—or in more general medical and scientific fields—is not only interesting, but necessary to uncover “systemic deficits” that may be ameliorated with a “cultural and macroscopic organizational-driven change” (176).

### 3.7. Country affiliations of first authors

Table 1 shows China with 69 first-authored publications followed by the USA with 35; these two have over one-half (55.0%) with the other 85 publications distributed over 28 countries in decreasing number of publications. Conspicuously absent are first-authored papers from the UK, Germany, Japan, and the Netherlands—countries which are consistently among the topmost productive countries in pain-related research (35, 40) and well-versed in bibliometric research (238–240).

While some researchers have published several bibliometric papers on pain in general and on specific aspects of pain using similar approaches (23, 35, 39, 49, 69), the large majority of first authors have published only one or two B/P papers. So, it appears to us that rather than arising from an institutional decision, the choice of using bibliometrics for studying the pain literature can mainly be interpreted as a desire of researchers made possible by colleagues with bibliometric knowledge and thereby increase the publication of bibliometric studies in non-bibliometric journals.

Furthermore, the domination of China over the USA first-authored papers in our study parallels a similar trend in the *PubMed* database over all biomedical publications: a search in March 2022 using only two parameters—*China* or *USA* in the “Affiliation” field and *Bibliometrics* in the “Title/Abstract” field—resulted in 454 documents for China and 268 for the USA.

### 3.8. B/P studies using *classica*l analysis

Each of the B/P papers that developed a general or classical analysis of the pain literature (Tables 2, 3: Group A) can be characterized through four main parameters:

(1) The first parameter is related to the type of pain considered. We note that in most of the studies (50 of 143), pain is considered without any restriction on the origin or the nature of pain (33, 35, 73, 85). The B/P papers that focus on specific types of pain are distributed as followed: studies on low back pain/back pain (*n* = 15), headache/migraine (*n* = 14), chronic pain (*n* = 12), fibromyalgia or neuropathic pain, each with 10 papers, cancer pain (*n* = 9), postoperative pain (*n* = 8), and a few on other specific types of pain such as trigeminal neuralgia (153), labor pain (90), postoperative pain (98), or psychological pain (94).
(2) The second parameter indicates the targeted population. The general population is targeted for most B/P investigations (25, 30, 76); however, several studies have restricted their population to patients with cancer (100, 126), diabetes (130), some critical illness (65), or depression (102).
(3) Another important parameter is the geographical limit of the populations studied. While most B/P papers have no geographical limits (54, 68), several studies are restricted to specific regions and/or geographical or economic situations: continent (51), sub-continent (96), set of countries (29, 98), country (28, 37), or institution (45).
(4) The last parameter specifies if the B/P studies highlight pain and its management or neither. Most of the B/P studies do not analysis the pain literature to this level of detail (116, 122); but, when this is the case, studies on the effects of acupuncture on pain dominates (*n* = 17), followed by studies involving drugs and analgesics (*n* = 12). Studies including other therapeutics such as alternative medicines (112), or complementary therapeutics (32) were also found.

To focus on an even more specific subset of the pain literature, other particulars are sometimes added such as, fRMI studies on acupuncture analgesia (101), or analysis of comorbidity in pain and depression (79), pain and cancer (114), or pain and inflammation (115).

A close observation of Tables 2, 3 (Group A) reveals that over time, B/P investigations have become more and more specialized in their aims and objectives: 53.7% (36 of 67) of the B/P literature from 1993 to 2019, and 78.9% (60 of 76) since 2020.

Bibliometrics analysis of pain research literature focusing on animals was the topic of interest in only a few studies (27, 42, 55, 186). As numerous improvements have been made during the last decade to provide more efficient and adaptable animal models of pain (241), the continuation of studies considering the ethical problems inherent in pain research (242) and bibliometric studies should proceed: firstly to quantify the evolution of the scientific community regarding the management of pain in animals, and then to globally enhance the quality and reliability of experimental research (55); and secondly to quantify the basic research on pain according to prescribed animal models.

### 3.9. Publishing *pitfall*s in B/P papers

Papers aimed at attracting the attention of readers to potential publishing pitfalls in science and medicine, including B/P studies, are shown in Table 4 (Group B).

Of particular interest in academia are preliminary or ongoing studies given orally or presented as poster papers at Congresses and their subsequent extension (or not) as articles in peer-reviewed journals. Two studies concern the abstracts presented at the *13th World Congress on Pain* sponsored by the *International Association for the Study of Pain* (IASP) in 2010 at Montreal, Quebec, Canada: one revealed that the overall “*publication rate*” (from Congress abstracts to full-text published papers) was 27.5% with variations among countries ranging from 12% for Switzerland to 38% for China (172); the second showed that just 52% of the abstracts dealing with Randomized Controlled Trials (RCTs) were later published as full-text papers (175).

Another study of papers presented during the *9th Brazilian Congress on Pain* held in 2010 at Fortaleza, Ceará, Brazil showed that only 8.9% appeared later as full-text papers (243). Perhaps the higher *publication rate* for Brazil (22%) of papers given at the IASP Congress at Montreal also held in 2010 might confirm the importance to authors for international visibility (172). Explanations generally given for “non-publication” include lack of time to prepare the manuscript for publication, lack of support from co-author(s), and time consumed by other ongoing studies (162). Additionally, there can be a lack of confidence in the quality and/or study design of the paper as well as the discovery of existing published papers with similar results: the former perhaps resulting from questions and discussions following either the presentation of papers or the viewing of poster papers. It is worth noting that the post-Congress period considered for eventual journal publications was 2 years for De Oliveira (162), 6 years for Saric et al. (175) and 7 years for Akkoc (172); therefore, comparing the results of the three studies is somewhat limited.

Another area of concern is the agreement between the *content* of the Congress abstract and its subsequent published full-text paper. An analysis of abstracts presented at various World Congresses of Pain revealed that a non-trivial percentage of the “*abstract-publication pairs*” have discordances: 31% for RCT abstracts; a high of 79% for abstracts reporting preliminary results (175); and 40% for systematic review abstracts (180). Such large discrepancies between abstract-publication pairs were noted in a recent editorial by Puljak and Saric positing if researchers should trust (and therefore cite or reference) abstracts from pain conferences; their studies have shown that “conference abstracts presenting the highest levels of evidence at the largest global pain congress are not necessarily dependable” (244).

In a similar vein, two recent bibliometric studies pinpointed discrepancies between the content of Congress abstracts and that of their full-text manuscripts. The studies concern low back pain papers obtained from the Physiotherapy Evidence Database (*PEDro*): one focusing on clinical trials (177) and the other on systematic reviews (178). The authors concluded that Congress abstracts of clinical trials and systematic reviews on low back pain were incomplete, show evidence of *spin* (overemphasis of beneficial effects), and were inconsistent with their full-text equivalents. Additionally, upon further scrutiny of published systematic reviews, three out of four were found to have “critically low” methodological quality (177, 182).

Finally, it is worth noting that despite the *Preferred Reporting Items for Systematic Reviews and Meta-Analyses* (PRISMA) 2020 Checklist (245) and its precursor guideline published in 1999 (246), the content of many low back pain systematic reviews and/or meta-analysis needs to be read with caution (167, 177, 180).

Occasionally investigated is the presence (or absence) of impact factor bias of papers published in pain journals. When testing the hypothesis that “*studies with positive results are more likely to be published in journals with greater impact factor when compared with articles with negative or inconclusive findings*” on RCTs published in pain journals, Mukhdomi et al. found no “impact factor bias in the pain literature across many journals over many years” despite differences in parameters, such as: origin of the data or size of the sample (181); presence of stated hypothesis or sponsorship funding. In contrast, a decade earlier De Oliveira found “publication bias in the anesthesiology literature especially in higher clinical trial impact factor journals” (162).

Even if the results of Mukhdomi et al. (181) are interpreted in the context of their limitations (a short time span, 2012–2018, and only nine pain journals considered), it is an encouraging sign for researchers and clinicians to submit “negative or inconclusive findings” to high impact journals. It can also be viewed as a positive stimulation for developing similar studies by broadening some criteria, such as: including non-pain-focused journals, extending the period of investigation, or including document types other than RCTs.

Four other papers that concern methodological approaches used in pain research complete this section:

(1) Yim et al. observed a high rate (79%) of misuse of statistics in papers published in the *Korean Journal of Pain* (161). This misuse rate is higher than those observed in other journals (247); later, the *Korean Journal of Pain* published a review paper presenting statistical issues related to pain research and describing some recommendations and suggestions (248).
(2) The second paper presents the characteristics and risks of bias when developing RCT studies, especially when the focus is on acupuncture and analgesia (166).
(3) In a short communication paper, some authors reported *spin* in various sections (Title, Results, Conclusion) of RCT articles published in pain and general anesthesia journals (183).
(4) The last paper is an assessment of reporting biases in RCT publications of first-line drugs for neuropathic pain (187).

These different pitfalls are not exclusive to pain research (166, 249–251) and most of them can be viewed as belonging to a set of harmful research practices referred to as *spin* or the misrepresentation and distortion of results in the scientific literature (252). The few B/P papers that noted the presence of *spin* and other pitfalls indicate the willingness of the pain community to keep rigorous standards, as illustrated by the continuous work developed by the team of R.H. Dworkin at the University of Rochester Medical Center (253–255).

Another publishing pitfall in the pain literature used a case-controlled approach and provided a 10-year follow-up of citations to retracted papers authored by Scott S. Reuben, an anaesthesiologist and pain researcher; Reuben was convicted of data fabrication and 25 of his papers were finally retracted—the highest number of retractions to date (188). The study showed that “invented or falsified data” continues to be cited over a decade later; this in turn leads to distortion (most likely) of results obtained by researchers of citing papers. The magnitude of Reuben's scientific fraud has been likened to the financial scandal of Madoff (http://www.elmundo.es/elmundosalud/2009/03/20/dolor/1237574917.html). Although the study highlights only one researcher, it illustrates that the field of pain research is not immune to fraudulence and inappropriate scientific behavior. With the development of specific databases to report retractions of scientific papers such as *Retraction Watch* (https://retractionwatch.com/), we encourage interested researchers to use bibliometrics to investigate the field of “retracted pain publications” as it could be used to track the spread of fraudulent papers in the pain literature; such an investigation was done for oncology (256). This would help pain researchers to remain vigilant in their analysis of the literature and to be aware of suspect articles.

### 3.10. *Miscellaneous* applications of bibliometrics in the B/P literature

If most B/P papers either focus on a quantitative description of the pain literature (Tables 2, 3: Group A) or use bibliometrics to highlight pitfalls in publishing (Table 4 Group B), a small number of papers with specific or unique aims have been published recently (Table 5 Group C). These 14 papers are characterized by the diversity of their: origins (from seven countries); publication format, e.g., *Letter* (200), *Correspondence* (192), *Original Article* (197–199), *Review* (196), or *Virtual Project* (195); topics—the analysis of pain literature through an Altmetric prism (190, 191, 199, 200), the development of a new bibliometric indicator to predict the success of an analgesic (189), the quantification of topics of existing pain research subject areas using natural language processing (198), the assessment of the evolution of the representation of pain in the brain for more than four decades using literature mining tools (194), etc. These studies provide evidence of the efficiency of bibliometrics for deciphering the pain literature and contributing to the knowledge of pain and its diffusion beyond the world of classical scientific publishing.

### 3.11. *Evolution* of bibliometrics in the B/P literature

In our study, the first paper quantifying the pain research literature was a two-page *Letter* published in 1993 which highlighted the distribution (by age groups) of pediatric pain publications in the 1980s. In this *Letter-to-the-Editor*, data are presented in a conventional table (14). Then, for about 20 years, after a few editorials highlighted the importance of conducting bibliometric investigations of the pain literature (257, 258), B/P researchers used standard “automated productivity applications” (e.g., Microsoft 365, formerly Office, including Word, Excel, and PowerPoint) and displayed data derived from their B/P analysis using, for example, Microsoft Office to create tables, charts, graphs, world/country maps, etc. In these papers, results are sorted and classified, in such a way as to provide the reader a simple but easily interpretable picture of the results and of the message proposed by the authors (see most of the papers in Tables 2, 3 published from 1999 to 2016). During the last decades, along with the growing importance of bibliometrics and advances in digital technologies, software packages dedicated to improving bibliometric investigation and data visualization have emerged (259); these tools have been used extensively in the analysis of pain-related literature. In 2016–2021, over one-half (56.1%) of the 73 B/P studies providing general bibliometric analysis (Tables 2, 3) used a software visualization tool (SVT). Among the different software visualization tools available, *CiteSpace* was used most often (24 papers), followed by *VOSviewer* (10 papers), *HistCite* (two papers); *BibExcel*, *Bibliometrix*, *BICOMB*, *Publish or Perish*, and *Word Cloud* (each with one paper). Of these 41 papers, four used two SVTs, and one used three SVTs. Detailed descriptions and reviews of the most relevant software visualization tools are available in several papers (1, 259, 260).

### 3.12. *Anomalies* in the B/P research literature

Throughout our analysis of the B/P literature, we found irregularities that occurred frequently enough to deserve the attention of readers:

(1) The date range of papers analyzed (e.g., 2010–2020) are often fuzzy (or incomplete) rather than inclusive; that is, retrieval took place before the closing date; see for example (59, 67, 89, 99, 101, 103, 108, 128, 134, 144, 149).
(2) In several studies, the retrieval date is approximate and exceeds the date range specified knowing that a delay of several weeks (months or longer) is needed to update the databases used such as *PubMed*, *Web of Science*; see for example (80, 85, 131, 133, 135, 152, 153, 155). Although this anomaly may not have serious consequences on the overall message of a study, such imprecision may introduce bias in the quantitative evaluation and consequently in its interpretation.
(3) Some papers using world map visualization tools in which a color scale is used to match each country with the number of pain publications indicated, produced some discrepancies between the numbers in the map and the numbers in the text; see for example (79, 82, 103, 111, 113, 114). Additionally, in the world maps of several studies, some countries are overlooked and hence, not displayed (82, 103, 116).
(4) In some studies, the data displayed in figures are illegible; see for example (79, 82, 88, 101, 111, 112, 114, 115, 130, 138–140, 149–151, 153).
(5) Several studies displayed figures with redundant information; for example, the number of papers, the number of citations, and the number of citations by papers; see for example (78, 79, 82, 88, 102, 111, 113–115, 119, 130, 138, 149).
(6) In many papers, the reader has to become familiar with specific indexes used in graph theory such as “centrality, closeness, betweenness, silhouette, strength, log-likelihood ratio”: these terms may be defined (124); at other times, only superficially introduced—see for example (67, 80, 88, 89, 92, 100, 101, 103, 105, 108, 112, 130, 137); and sometimes not defined at all (94, 97, 117, 123, 134, 135, 138–141, 150).
(7) In many studies where figures presented are from software visualization tools composed of graphs with nodes representing a variable (e.g., country, author, institution) interconnected to other nodes according to intensity or proximity, the reader is confronted with:
  • a surfeit of figures—see for example (78, 79, 82, 88, 89, 93, 98, 101–103, 108–115, 118, 123–125, 130–132, 134, 135, 137–142, 149–151, 153);
  • a surfeit of figures with a melange of colors for nodes, links, labels which makes the message hard to interpret and understand (93, 105, 131, 139);
  • links *without* nodes or labels (67, 79, 80, 82, 88, 89, 94, 102, 103, 105, 111–115, 117, 118, 130, 132, 134, 138, 140, 142, 149, 150); and
  • links *with* nodes but *without* labels or vice versa (67, 78–80, 82, 88, 89, 100, 102, 103, 105, 108, 111–115, 117, 118, 125, 130–132, 134, 135, 139–142, 148–150, 153).

Along with the recurring anomalies listed above, other observations of non-standard scientific practice include, inter alia: lack of agreement between information displayed in figures and tables (e.g., 112); inadequate or omission of key search parameters, such as the topic, in the Methods section (e.g., 58); absence of citations in the Discussion section (e.g., 152); cited references in the text or tables either omitted or incorrectly listed in the Reference section (e.g., 67, 97, 118, 141); non-conforming in-text citation practice (e.g., 119); countries often misnamed (e.g., England for the UK), appear twice (e.g., Germany) or mistaken as states (e.g., TX, NJ, CA for the USA). The last observation may be due to the combining of two or more databases (data sources) with varying granularities in a “field” designated as “country”.

Finally, we would encourage readers to be vigilant when reading past, present, or future bibliometric studies of a subject literature such as “pain”. We would also like to encourage authors and editors to take extra care in writing and editing papers before publication; this practice will certainly lighten the burden on readers and (perhaps) increase the profile of such publications and their authors through higher citation counts in databases as well as higher Altmetric scores.

## 4. Conclusion

This study presents a large view of the numerous bibliometric investigations on the pain literature developed during the last 30 years. Most of these studies provide general descriptions of the pain literature with filters adapted to the objectives of each study: selecting a type of pain; focusing on a geographical (world, continent, country) population with or without any specific health-related status; or highlighting pain therapeutics. Other B/P studies are dedicated to reveal or analyze publishing pitfalls existing in the pain literature, and a few papers include some miscellaneous applications. Since the number of B/P papers has dramatically increased in the last years, providing useful information for the pain medical and scientific community, it is recommended that readers be cautious when reading and interpreting the results of B/P papers.
